# Linking Genes to Molecules in Eukaryotic Sources: An Endeavor to Expand Our Biosynthetic Repertoire

**DOI:** 10.3390/molecules25030625

**Published:** 2020-01-31

**Authors:** Jack G. Ganley, Emily R. Derbyshire

**Affiliations:** 1 Department of Chemistry, Duke University, 124 Science Drive, Durham, NC 27708-0346, USA; john.ganley@duke.edu; 2 Department of Molecular Genetics and Microbiology, Duke University Medical Center, 213 Research Drive, Durham, NC 27710, USA

**Keywords:** natural products, biosynthetic gene clusters, eukaryotes, algae, animals, apicomplexans

## Abstract

The discovery of natural products continues to interest chemists and biologists for their utility in medicine as well as facilitating our understanding of signaling, pathogenesis, and evolution. Despite an attenuation in the discovery rate of new molecules, the current genomics and transcriptomics revolution has illuminated the untapped biosynthetic potential of many diverse organisms. Today, natural product discovery can be driven by biosynthetic gene cluster (BGC) analysis, which is capable of predicting enzymes that catalyze novel reactions and organisms that synthesize new chemical structures. This approach has been particularly effective in mining bacterial and fungal genomes where it has facilitated the discovery of new molecules, increased the understanding of metabolite assembly, and in some instances uncovered enzymes with intriguing synthetic utility. While relatively less is known about the biosynthetic potential of non-fungal eukaryotes, there is compelling evidence to suggest many encode biosynthetic enzymes that produce molecules with unique bioactivities. In this review, we highlight how the advances in genomics and transcriptomics have aided natural product discovery in sources from eukaryotic lineages. We summarize work that has successfully connected genes to previously identified molecules and how advancing these techniques can lead to genetics-guided discovery of novel chemical structures and reactions distributed throughout the tree of life. Ultimately, we discuss the advantage of increasing the known biosynthetic space to ease access to complex natural and non-natural small molecules.

## 1. Introduction

Nature’s ever-growing repository of chemical structures and reactions continues to shape the fields of chemistry and biology. Of particular note is the impact of natural products in modern medicine. It is estimated that anywhere from 50 to 70% of all clinically approved drugs are natural products, natural product derivatives, or inspired by natural products [1]. Specifically, 51% of anticancer new chemical entities (NCEs) and 59% of antibiotic NCEs from 1981 to 2014 were natural products or directly derived from there [1]. As nature’s small molecules are optimized through years of evolution, their affinity to their molecular targets is often exquisitely high. Thus, the discovery of natural products, especially with known targets, affords privileged chemical scaffolds for drug discovery and development [2].

Along with inspiration for drug design, natural product research presents critical questions about evolutionary ecology from individual species to entire biospheres [3,4]. At the micro-ecology scale, inter- and intra-species interactions are often mediated through small molecules that can grant evolutionary advantages to the producer [5]. Understanding these interactions can provide insight into complex biological systems and potentially provide a means to precisely manipulate these microecosystems [6]. This is epitomized by human gut microbiome studies, where understanding gut dysbiosis has led to prophylactic and responsive therapies [7]. On a larger scale, natural products can affect entire ecosystems. For example, harmful algal blooms releasing toxic small molecules can decimate entire aquatic ecosystems and, consequentially, influence socioeconomic systems [8].

Due to the inarguable utility of natural products, researchers have questioned the scope of structurally distinct molecules that can be produced by nature. While over the past 60 years there has been an attenuation in the natural product discovery rate and overall structural complexity, structurally-intriguing metabolites with diverse biological activities are continually disclosed each year [9]. Reports of unique structures are often identified from a broad phylogenetic range of organisms, ultimately suggesting that a vast proportion of nature’s chemical space remains undiscovered [9]. While typical natural product producing organisms still generate novel chemical structures, rediscovery has presented an obstacle [10]. One way to circumvent this is to innovate discovery methods and explore phylogenetically diverse organisms from various environments [9].

Over the past 20 years, genomic, transcriptomic, and bioinformatic advances have ignited natural product discovery approaches. Genetics-guided techniques, including genome mining, afford considerable advantages [11], especially in efforts to explore uncharted chemical space [12], or discovering metabolites with predicted molecular targets [13]. With a plethora of genome sequencing data publicly available [14], researchers can use bioinformatic platforms, such as antiSMASH [15], PRISM [16], and many others [17,18,19], to identify BGCs and test various hypotheses. Not only has this approach led to the identification of novel structures but it has also helped answer basic science questions surrounding gene cluster activation [20], self-resistance [21], and horizontal gene transfer [22], among others. Additionally, advances in automated DNA synthesis has greatly facilitated genome mining expeditions [23] and they provide an efficient auxiliary to molecular biology-based assembly of BGCs [24]. As the price per base for DNA synthesis continues to decrease [25,26], purchasing large BGCs of interest becomes less financially burdensome, enabling expression of BGCs in heterologous hosts.

**Figure 1:**
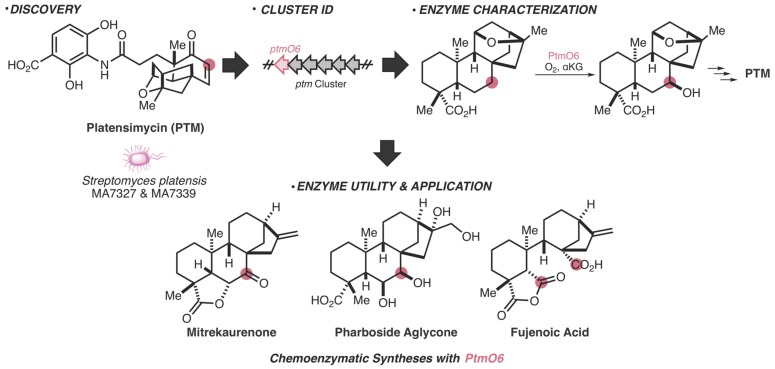
Workflow from natural product discovery to enzyme utility in accessing complex molecules. The example shown illustrates the chronological order of research on platensimycin (PTM), from molecule discovery [29] to biosynthetic gene cluster (BGC) identification [30] to enzyme characterization [31]. This directly enabled the use of an enzyme from this pathway (PtmO6) in the synthesis of the complex natural products mitrekaurenone, pharboside aglycone, and fujenoic acid [32]. Pink dots represent carbons that are functionalized by PtmO6.

Meticulous investigation of how nature synthesizes molecules has not only inspired retrosynthetic design [27] but has also uncovered synthetically useful enzymes to facilitate previously elusive reactions [28]. Within bacteria, the workflow from the initial discovery of a natural product or BGC to chemoenzymatic functionality is versatile and can be approached from different paths. Biosynthetic investigation of the bacterially produced antibiotic, platensimycin, is one of many examples that displays how nature’s ability to manufacture molecules can directly enhance our synthetic tool kit (Figure 1). Through molecule discovery [29], gene cluster identification [30], and subsequent biosynthesis elucidation [31], an enzyme from this pathway has been successfully utilized in multiple chemoenzymatic total syntheses of difficult-to-access natural products [32]. In an ideal situation, researchers will have a specific application in mind and will be able to use bioinformatics to identify enzymes with the desired utility.

Tremendous chemical and enzymatic diversity exist outside of bacteria and fungi, however, a multitude of technical difficulties impede discovering and harnessing these resources. A first step towards this goal is unlocking the biosynthetic potential of phylogenetically diverse organisms and continuing efforts to improve bioinformatics, genetics, enzymology, heterologous expression systems, and more in eukaryotes. Advancing available genome mining techniques in non-fungal eukaryotes faces a myriad of technical obstacles, especially due to the vast phylogenetic disparities between organisms. Despite this, diligent and creative approaches are enabling the field to unlock the biosynthetic potential of eukaryotes. Herein, we explore the recent advances in linking genes to previously known molecules, as well as genetics-guided approaches to find new molecules in phylogenetically diverse organisms.

## 2. Current State of Connecting Genes to Molecules and Evaluation of the MiBIG Repository

To gauge the state of connecting molecules to their BGCs in eukaryotes, we chose to analyze the biological sources of characterized BGCs. The Minimum Information about a Biosynthetic Gene Cluster (MiBIG) repository [33], an extension of the Genomic Standards Consortium (GSC) [34], has led the way in standardizing and compiling BGCs. MiBIG is driven by community-based submission of newly characterized biosynthetic gene clusters where researchers can submit partially or fully characterized BGCs. MiBIG additionally includes evidence connecting the gene(s) to the resulting molecule(s) (i.e., knock out studies, heterologous expression, etc.). The platform is integrated with bioinformatic tools such as antiSMASH, allowing users to compare query sequence clusters to previously characterized BGCs. Although there is undoubtedly data absent from MiBIG, we found it to be a useful reference to evaluate the current state of the field in regard to connecting BGCs to natural products.

**Figure 2:**
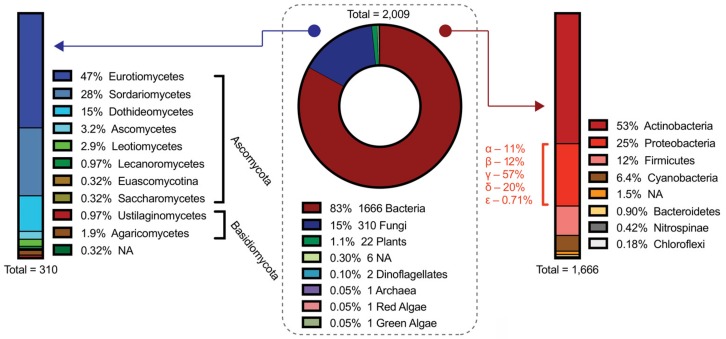
Analysis of the Minimum Information about a Biosynthetic Gene Cluster (MiBIG) repository [33]. The pie chart (center) shows the natural product source for all 2009 BGCs deposited on the MiBIG repository. All 1666 bacterial BGCs are further categorized by phylum (right) and Proteobacteria are further categorized by class. All 310 fungal BGCs are further categorized by phylum and class (left).

After analyzing all 2009 of the BGCs within the MiBIG repository (as of November 2019), it is unsurprising that bacteria have the most characterized clusters at ~83%, followed by fungi at ~15%, and plants at ~1%. Within bacteria, further examination shows that ~53% belong to the Actinobacteria phyla, followed by Proteobacteria (~25%), Firmicutes (~12%), and Cyanobacteria (~6%) (Figure 2). Bacteria, particularly actinobacteria and proteobacteria, have a tremendous capacity for the production of complex natural products. In some cases, bacteria have accumulated 40 to 60 BGCs into their genome, which often accounts for upwards of 20% of their genomic real estate [35]. This astounding attribute is part of the reason bacterial sources are gold mines for natural product discovery, especially as we continue to innovate novel mechanisms of unveiling cryptic metabolites [36].

Of note, inherent genomic and transcriptomic differences exist between bacteria and eukaryotes that can influence the success of identifying BGCs. Bacteria typically organize functionally related genes into operons that are transcribed as a polycistronic mRNA and, subsequently, translated into multiple separate proteins. Conversely, eukaryotic genes are typically monocistronic, where mRNA is translated into a single protein product. Over the past half century, genomic clustering of functionally-related genes has been observed in eukaryotes [37]. Although these metabolic gene clusters in eukaryotes are nearly always monocistronic, there are some intriguing similarities to bacteria, which were recently summarized at length by Osbourne and colleagues [38].

Among eukaryotes, fungi, and plants are prolific sources of natural products [39,40]. For thousands of years, humans have used fungi and plants as sources of traditional medicine [41,42] due to their propensity to synthesize bioactive secondary metabolites, yet we are still relatively unfamiliar with how these molecules are assembled. Although fungi have the most characterized eukaryotic BGCs with 310 deposited on MiBIG, our knowledge of fungal secondary metabolism is still limited considering over 1500 compounds were isolated from fungi between 1993 and 2001 alone [43]. Plants have even fewer characterized BGCs despite a more diverse and complex secondary metabolome than fungi. Innovative techniques to discover, express, and characterize these eukaryotic BGCs have paved the way for future biosynthetic revelations and synthetic utilities. Discovery advances for fungi and plants are not discussed herein, however, they have been aptly highlighted in recent reviews [38,44,45]. While plants and fungi are quintessential eukaryotic natural product producers, sources from diverse lineages, including algae [46], dinoflagellates [47], and even animals [48] have displayed the capacity to produce complex natural products with a broad range of bioactivities.

## 3. From Molecules to Gene Clusters in Dinoflagellates and Algae

With nearly 30,000 marine natural products (prokaryotic and eukaryotic) described to date, including 1490 novel structures from 2017 alone [49], there is little doubt surrounding the prolificacy of marine sources for natural product discovery. While identification of the producing organism of many marine natural products is convoluted due to microbial counterparts [50], there is still evidence of de novo biosynthesis in many marine non-fungal eukaryotes [51,52]. Two exemplary lineages of marine-natural product producers are algae and dinoflagellates. In particular, these marine organisms are well-known for their ability to produce small molecules including toxins, pigments, unique lipids, and a variety of other natural products [47,53,54,55]. Despite many of the structures of algal and dinoflagellate molecules being known for decades, connecting them to their enzymatic machinery is in its infancy.

**Figure 3:**
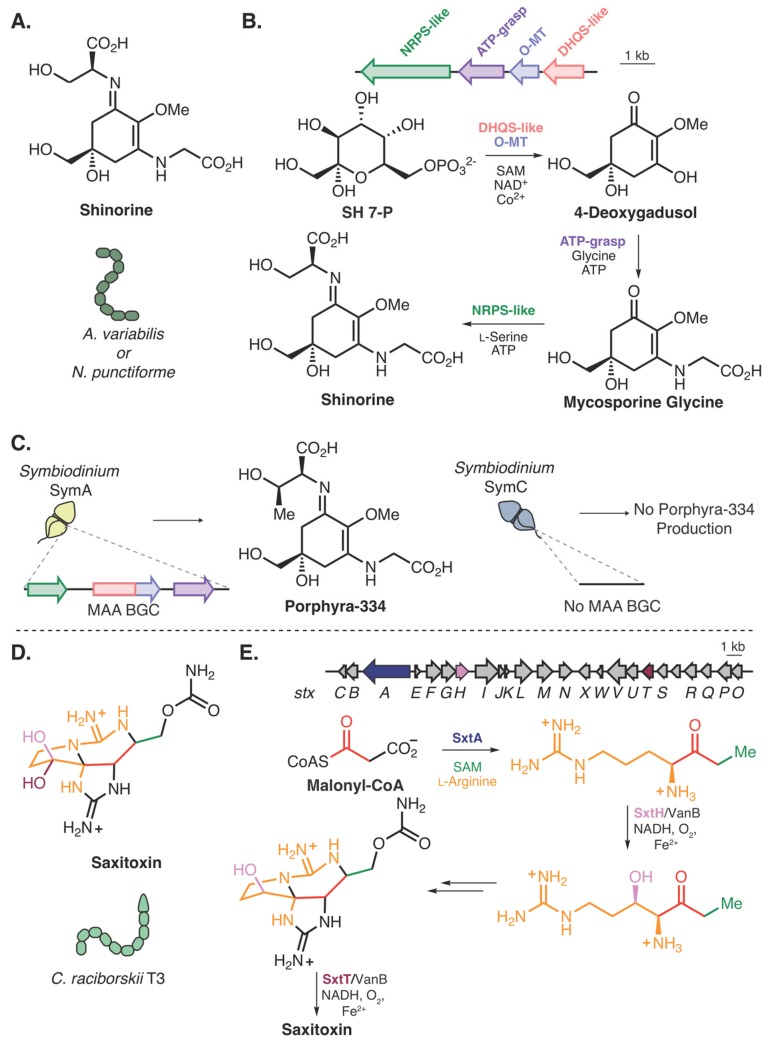
Natural products present in both cyanobacteria and marine eukaryotes. (**A**) Structure of the mycosporine-like amino acid (MAA) shinorine produced by *Anabaena variabilis* and *Nostoc punctiforme*. (**B**) Biosynthesis and BGC of shinorine from *Anabaena variabilis* and *Nostoc punctiforme* [59]. SH 7-P; Sedoheptulose 7-phosphate. (**C**) The MAA Porphyra-334 is produced in the dinoflagellate *Symbiodinium* SymA and the BGC was identified, while *Symbiodinium* SymC does not produce MAAs and no BGC is found within the genome [56]. (**D**) Structure of saxitoxin produced by *Cylindrospermopsis raciborskii* T3. (**E**) The BGC for saxitoxin (*stx*) and resolved biosynthetic steps [68,69].

Among the BGCs deposited on the MiBIG repository, four clusters belong to organisms from algae or dinoflagellate organisms, including two BGCs responsible for the production of mycosporine-like amino acids (MAAs) (Figure 3A) (BGC0001678, and BGC0001883) from the dinoflagellate genus, *Symbiodinium* [56]. MAAs are naturally occurring sunscreens [57] that absorb ultraviolet light and have been found to be produced by cyanobacteria [58,59], bacteria [60], fungi [61], coral [62], and even fish [63]. A biosynthesis for this class of natural products was described by Walsh and Balskus, wherein heterologous expression of a small BGC from cyanobacteria species and subsequent in vitro reconstitution of the enzymes illustrated conversion of the primary metabolite, sedoheptulose 7-phosphate, to MAAs [59] (Figure 3B) (a different biochemical route exists for marine invertebrates [63]). While characterization of MAA biosynthesis has not been investigated biochemically in dinoflagellates, the discovery in cyanobacteria facilitated the bioinformatic assignment of this BGC in *Symbiodinium* dinoflagellates. Within *Symbiodinium*, production of MAAs varies between species. A recent study compared genomes of two species of *Symbiodinium*, one that produces the MAA porphyra-334 and another that does not, and identified a homologous BGC within the producing strain, which was absent in the nonproducing strain [56] (Figure 3C).

There are limited examples of molecules that are produced by both cyanobacteria and marine eukaryotes, but when this occurs it provides an approach to generate hypotheses about eukaryotic natural product biosynthesis. For example, some paralytic shellfish toxins (PSTs), such as saxitoxin (Figure 3D), are produced by both cyanobacteria and dinoflagellates [64,65,66]. While a putative gene cluster for saxitoxin in cyanobacteria was identified in 2008 [67], biochemical confirmation was lacking until recently. Narayan and coworkers have established core biosynthetic steps including the biosynthesis of the main precursor to saxitoxin formation [68], C–H hydroxylations [69] (Figure 3E), and detoxification steps [70]. This molecule is found within several species of dinoflagellates, but only a few studies have biochemically tested enzymes related to PST biosynthesis within these organisms [71,72]. Moreover, full-length BGCs for these toxins have yet to be identified in dinoflagellates, likely due to the difficulty in assembling and sequencing the large genomes; however, homologs to *sxtA*, *sxtG*, and *sxtB* have been identified from transcripts in the dinoflagellate, *Alexandrium tamarense* [65].

**Figure 4:**
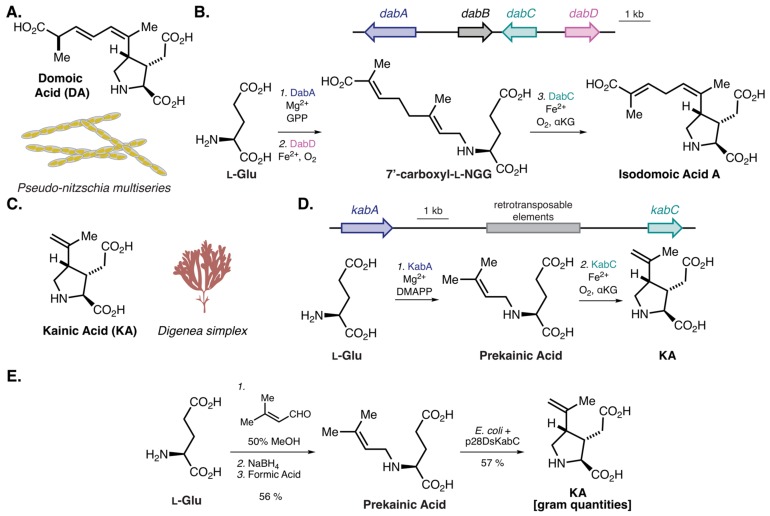
Natural products and their biogenesis from marine eukaryotes. (**A**) Structure of DA from the diatom *Pseudo-nitzchia multiseries*. (**B**) The domoic acid (DA) BGC from *Pseudo-nitzchia multiseries* and the biosynthesis to isodomoic acid A [79]. l-NGG; *N*-geranyl-l-glutamic acid. (**C**) Structure of kainic acid (KA) from the seaweed *Digenea simplex*. (**D**) The KA BGC from *Digenea simplex* and the biosynthesis of KA. (**E**) Semisynthetic route to KA by Moore and colleagues [80].

In addition to saxitoxin, there are many other algal bloom-associated toxins produced by algae and dinoflagellates [73]. Harmful algal blooms, typically caused by either cyanobacteria, dinoflagellates, or diatoms, have tremendous ecological and economic impacts and result in large marine animal mortality [74]. Of particular note are diatoms from the genus, *Pseudo-nitzschia*, which often synthesize the toxin, domoic acid (DA) [75] (Figure 4A). DA, whose structure was fully elucidated in 1966 [76], is an excitatory glutamate receptor agonist that can cause amnesic shellfish poisoning in humans and marine animals [77,78]. Despite the structure being known for 54 years, the biogenesis of DA remained elusive until 2018. To identify candidate biosynthetic genes, Moore and colleagues conducted a differential transcriptomic analysis of the DA-producing diatom *Pseduo-nitzschia multiseries* in DA-stimulating growth conditions as compared with conditions where DA production is limited [79]. From this analysis, four of the top eight upregulated genes were found to be clustered within the genome, consisting of a terpene cyclase (*dabA*), a hypothetical protein (*dabB*), a dioxygenase (*dabC*), and a cytochrome P450 (*dabD*) (Figure 4B). Through in vitro enzyme reconstitution with either heterologously expressed and purified proteins or yeast microsomes expressing candidate enzymes, a biosynthetic route to isodomoic acid was determined (Figure 4B). Currently, the final isomerization to DA remains elusive, but this work illustrates the utility of differential transcriptomics to determine candidate biosynthetic genes [79].

The work described above directly facilitated biosynthesis studies of the related ionotropic glutamate receptor agonist, kainic acid (KA) [80] (Figure 4C), produced by the tropical seaweed *Digenea simplex (Rhodophyta*/red algae) [81]. Since DA and KA have similar chemical structures, Moore and coworkers hypothesized DabA- and DabC-like enzymes were likely responsible for the generation of KA. Using recently developed single-molecule, long-read sequencing technology [82], which can generate >100 kilobase (kb) single reads, large genomic regions (N50 of 7 kb) of the *D. simplex* genome were sequenced and ultimately assembled. From this, a 12-kb (*kab* BGC) region was discovered that encodes a putative *N*-prenyltransferase (*kabA*) and a dioxygenase (*kabC*) (Figure 4D), which were subsequently expressed and purified in *E. coli*. In vitro reconstitution illustrated that the two enzymes were sufficient to synthesize KA from dimethylallyl pyrophosphate (DMAPP) and l-glutamate, validating the biosynthesis of KA in *D. simplex*. To generate a practical route to produce KA, Moore and colleagues synthesized the KA precursor prekainic acid via reductive amination and added this to KabC-expressing *E. coli.* Impressively, this strategy provided over 1 g of purified KA [80] (Figure 4E). Although there are multiple total syntheses of KA (over 70) [83], including scalable routes of at least six steps [84], this work illustrates the utility of harnessing biocatalysis to access complex molecules and provides a two-step route to an important and structurally complex molecule.

In spite of the inherent difficulties associated with identifying BGCs from algae and dinoflagellates, including inordinately long and complex genomes, lack of reference genomes, difficulty in separation of host- and metagenomic reads, and hurdles in collection and culturing, research over the past decade has connected the first BGCs to their respective molecules in these species. These BGC:molecule associations have already led to the use of an algal enzyme in a concise semisynthesis of a difficult-to-assemble marine natural product.

## 4. From Molecules to Gene Clusters in Animals

A compelling area of natural product research is the investigation of secondary metabolites produced by animals. Natural products arising from animals have been mistakenly attributed to a microbial counterpart, a dietary artifact, or a combination of the two [50,85]. But selected secondary metabolites from humans [86,87,88], insects [89,90], and sea urchins [91] have been connected to their cognate BGCs, and this number has been growing in recent years. A compelling review by Torres and Schmidt discusses many of the advances in animal biosynthesis [48], while additionally challenging the perceived notion that animals are not resources for natural products. To further expand upon this, we discuss some of the recent advances in animal natural product biosynthesis, including molecule- or phenotype-driven BGC discovery from both invertebrates and vertebrates.

**Figure 5:**
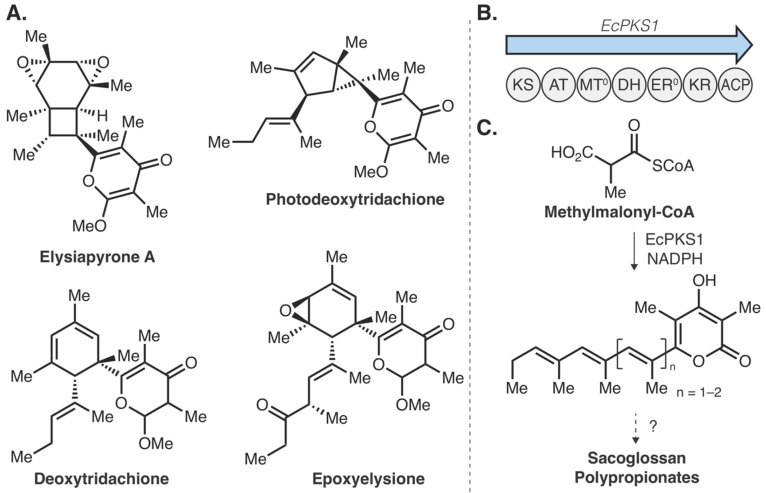
Sacoglossan sea slug polyproionate biosynthesis. (**A**) Representative structures of four polypropionates from *Elysia* spp. (**B**) Polypropionate synthase in *Elysia chlorotica* (*EcPKS1*) and its domain architecture. (**C**) In vitro reconstitution of EcPKS1 with methylmalonyl-CoA and NADPH led to the triene pyrone and tetraene pyrone, likely precursors to sacoglossan polypropionates [96].

Along with algae and dinoflagellates, marine invertebrates have proven to be lucrative sources of natural products. While many of these natural products are indeed produced by associated microbiome members [50], de novo production has also been illustrated in marine animals [92], including sea slugs [51], nudibranchs [52], sponges [93], corals [62,94], sea urchins [91], and bryozoans [95], to give some examples. With a lack in genomic and transcriptomic data, connecting these molecules to their associated gene clusters remains a challenge. The sacoglossan sea slugs provide an intriguing example of connecting a marine natural product to the responsible genes [96]. Sea slugs from the *Elysia* genus are a fascinating clade of mollusks that often partake in kleptoplasty, a phenomenon where the chloroplasts from consumed algae are maintained and used as an energy source via photosynthesis [97]. A recent discovery illustrated that the defense compounds kahalalides, found in *E. rufescens* and the green algae it consumes (*Bryopsis* sp.), are actually produced via endosymbiotic bacteria residing within the green algae [98]. The complex intra-species relationships employed in this system highlights the challenges of tracing molecules to their producing organism. *Elysia* mollusks also produce complex natural products de novo [51]. The sacoglossan polypropionates (Figure 5A) are complex natural products that are produced with fixed carbon via photosynthesis [51]. Isotopic labeling studies suggest the polypropionates are assembled through iterative construction of methylmalonyl-CoA building blocks, indicating the metabolites are fatty acid and polyketide derived [99]. To determine the biochemical machinery of the polypropionates, Schmidt and colleagues first sequenced the bacterial metagenome to discover putative polypropionate synthase genes. While no good candidates were identified from bacteria, they found potential polypropionate synthase genes by examining recently published *Elysia* spp. genomes and transcriptomes [100,101,102,103]. From *E. chlorotica,* a representative polypropionate producer, four genomic fatty acid synthase (FAS)-like genes were identified, two of which belonged to primary metabolism, while the remaining two were classified as animal polyketide synthases (PKSs, EcPKS1, and EcPKS2) (Figure 5B). Bioinformatic analyses suggested that EcPKS1 likely recognizes methylmalonyl-CoA as a substrate while EcPKS2 can use malonyl-CoA, therefore, EcPKS1 was investigated for polypropionate biosynthesis. Subsequent in vitro reconstitution of EcPKS1 with methylmalonyl-CoA and NADPH led to production of a triene pyrone and a tetraene pyrone (Figure 5C), the latter of which is consistent with the predicted precursor for tridachione polyprionate natural products [99]. Although tailoring steps (methylation, oxidation, and photochemical) and chain length-determining factors remain elusive, this work illustrates an excellent example of polyketide biosynthesis from an animal source and sets the stage for exploring marine animals as sources of structurally diverse natural products.

**Figure 6:**
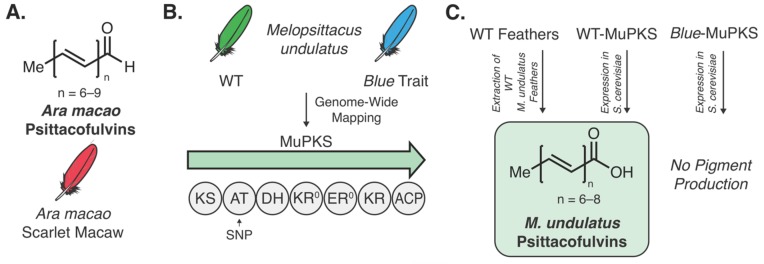
Polyene natural products and their role in pigmentation in birds. (**A**) Psittacofulvins isolated from the scarlet macaw. (**B**) WT feathers in *Melopsittacus undulatus* are green. The blue trait was mapped to a single-nucleotide polymorphism (SNP) located within the MuPKS. (**C**) Heterologous expression of MuPKS in yeast led to the production of the same polyene pigments found within WT feathers. Heterologous expression of the MuPKS with the blue trait SNP led to no pigment production [104].

Natural product biosynthesis is not limited to invertebrate animals. Examples of connecting genes to molecules have been illustrated in fish [63], birds [104], and humans [86,87,88,105]. Of particular note is the discovery of the genetic determinant of coloration in parrots [104]. While many bird pigments, such as carotenoids, are derived from dietary origins [106], psittacofulvins pigments, originally isolated from scarlet macaw parrots (*Ara macao*) (Figure 6A) [107] were hypothesized to be produced de novo based on the lack of pigments identified in blood and diet samples [108]. Budgerigar parrots (*Melopsittacus undulatus*) are known for their yellow-green coloration, however, after years of captivity a recessive Mendelian blue trait (i.e., blue feathers) has been observed [109,110,111,112]. As no genetic basis for this observed trait was known, Bustamante and coworkers conducted genome-wide association mapping and expression analysis to identify the genetic determinants of feather color [104]. From this, a single-nucleotide polymorphism (SNP) was identified within the acyltransferase domain of a putative type-I PKS (MuPKS) (Figure 6B). Heterologous expression of the WT MuPKS in yeast produced identical pigment metabolites to those seen in the WT feather extracts, while heterologous expression of the blue trait MuPKS (with the SNP) produced no such metabolites (Figure 6C). Additional phylogenetic analysis of animal PKS genes revealed a multitude of animals contain PKS genes with variable but similar domain architecture, prompting questions surrounding the evolutionary origin and purpose of animal PKS genes. To begin to address some of these questions, a PKS similar to MuPKS from a non-psittacofulvin producing bird (chicken, *GgPKS1*) was heterologously expressed in yeast. With *GgPKS1* overexpression the two previously identified psittacofulvins were produced as well as two other metabolites with unknown structures. This finding indicates that expression pattern differences, not the gene coding sequence, is responsible for psittacofulvin coloration and variation [104]. While this experiment answers important evolutionary questions, it also embarks upon a technically onerous research area, i.e., genetics-guided discovery of new chemical space in non-fungal natural product producers.

A great deal of chemical space awaits to be discovered within metazoans. Even in insects, the most phylogenetically diverse group of animals [113] and established natural product producers [114], a relatively small number of examples providing enzymatic or genetic evidence to connect insect-produced metabolites to their biosynthetic gene(s) exist [48,89,90,115,116,117,118]. With continued efforts to generate more genomic and transcriptomic data from diverse animals, more natural products with unique structures and bioactivities will undoubtably be discovered from these sources. Thus, a next logical progression of this field is genetics-guided studies to find novel chemical space in animals and other eukaryotes.

## 5. Genetics-Guided Discovery of Natural Products in Eukaryotes

As efforts persist to sequence and assemble genomes of organisms from across the Earth, bioinformatics will continue to identify regions that are predicted to produce novel chemical structures. As demonstrated with the chicken *GgPKS1* and numerous bacterial and fungal BGCs, many of these genes can be transcriptionally silent. Even in cases of transcriptionally active BGCs, isolating sufficient material for structural determination can be an arduous or unfeasible task. Further compounding the difficulty of genome mining in eukaryotes is the fact that biosynthetic genes do not always cluster within genomes. Clustering of functionally related genes does indeed occur within eukaryotic organisms [38], however in many cases related biosynthetic genes have disparate genetic loci [119,120].

**Figure 7:**
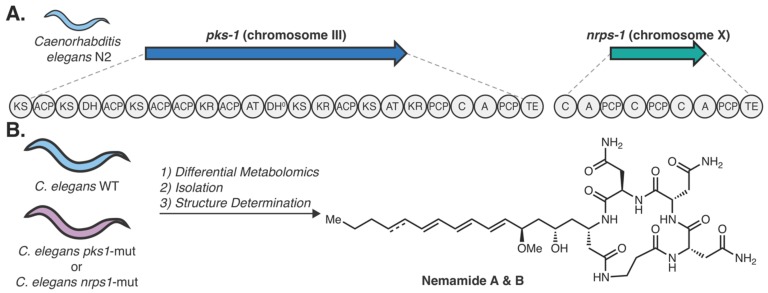
Nemamide biosynthesis in *Caenorhabditis elegans*. (**A**) Domain architecture of the *Caenorhabditis elegans* N2 PKS (*pks-1*) on chromosome III and the NRPS (*nrps-1*) on chromosome X. (**B**) Differential metabolomics approach using both *pks-1* and *nrps-1* mutated worms against WT worms to identify the nemamide metabolites, which were subsequently isolated and structurally characterized [119].

Butcher and colleagues were able to circumvent some of these hurdles to demonstrate an example of genetics-guided natural products discovery in eukaryotes [119]. Specifically, this study examined potential products associated with either a multi-modular PKS gene (*pks-1*) or an NRPS gene (*nrps-1*) encoded by the nematode *Caenorhabditis elegans* (Figure 7A) [121]. Despite the well-known ability of *C. elegans* to produce interesting secondary metabolites [122], multi-modular PKS and NRPS genes in metazoans are rare and had not yet been explored. Differential metabolomic analysis of the *pks-1* and *nrps-1* mutated worms against wildtype worms showed the same two metabolites were absent in both mutants. Together, this suggested that the PKS and NRPS work in tandem to synthesize the metabolites. The molecules, termed nemamide A and B, were subsequently isolated from large quantities of wildtype worms (50 L), which afforded small quantities of each (<80 µg). Through a combination of NMR, mass spectrometry, Marfey’s analysis, circular dichroism, and bioinformatics, the structures of the nemamides were determined (Figure 7B). Using a GFP transcriptional reporter strain for both *pks-1* and *nrps-1*, the genes were shown to be transcribed at all life stages of the nematodes and colocalized with canal-associated neurons. Further work illustrated that both *pks-1* and *nrps-1* mutants recovered from starvation slower than wildtype worms, suggesting a potential developmental role for the nemamides [119]. This work epitomizes the potential of genetics-guided natural product discovery in phylogenetically diverse eukaryotes, as it resulted in novel structures with intriguing bioactivities. Furthermore, these studies enable hypotheses about the role of these secondary metabolites in *C. elegans* biology.

There are many examples of BGCs from unique eukaryotes, some even with known biological functions, whose products are unknown. These represent prime candidates for future genetics-guided natural product discovery efforts. One such example involves biomineralization in various animals, including zebrafish (*Danio renio*) [123], Japanese rice fish (*Oryzias latipes*) [124], and sea urchins (*Hemicentrotus pulcherrimus*) [124]. In general, biomineralization is the process in which animals synthesize typically rigid chemical structures, often to stiffen or protect softer tissues. Examples of this range from vertebrate bones and teeth to calcium-based shells and coral reefs. In 2015, Hojo et al. investigated the biomineralization products otoliths from *O. latipes*. Otoliths are produced within vertebrate inner ears and aid in sensing motion, gravity, and balance [124]. They found that an *O. latipes* mutant unable to produce otoliths has a 9-nucleotide deletion within the ketosynthase domain of a putative PKS gene (*OlPKS*). In otolith-lacking zebrafish, similar mutations in homologous PKS genes have also been observed, suggesting a conserved function [123]. To validate the role of *OlPKS*, the entire gene was heterologously expressed in *Aspergillus oryzae* and the resultant organic extracts were sufficient to restore otolith formation in mutant *O. latipes*, suggesting the yet to be discovered PKS product(s) serves as an otolith nucleation factor [124]. Bioinformatic analysis of similar animal PKS genes identified a homolog within the sea urchin *H. pulcherrimus* (*hppks-2*). Using a morpholino knockdown strategy in *H. pulcherrimus*, echinoderm embryos with lowered *hppks-2* levels exhibited dramatically altered spicule formation, another biomineralization related structure. Together, these data suggest that many animal secondary metabolites, polyketides in particular, could have conserved functions that are understudied. Although the associated structures from *OlPKS* and homologs remain unresolved, *OlPKS* was successfully expressed in a fungal host, an auspicious result for genetics-guided natural product discovery in non-fungal eukaryotes.

Another group of organisms that is an excellent candidate for future genetics-guided natural product discovery is apicomplexan parasites. Apicomplexans are single-celled eukaryotic parasites that infect a multitude of organisms and typically reside within their host [125]. Of particular note are parasites from the *Plasmodium*, *Toxoplasma*, and *Cryptosporidium* genera that cause malaria, toxoplasmosis, and cryptosporidiosis, respectively. These organisms are phylogenetically related to red algae and dinoflagellates [126,127] and their potential as untapped sources of novel metabolites has been previously reviewed by our group [128]. Some secondary metabolites from these organisms have been associated with altering specific life cycle stages, including facilitating egress from the host cell [129] and potentially increasing transmission rates [130]. Remarkably, many of these organisms contain multi-modular type I PKS and FAS genes with unknown metabolites [128,131,132]. While there is not a definitively known function for the polyketide metabolites within these organisms, we [131] and others [133,134] have hypothesized structural roles in oocyst wall formation, analogous to polyketide-derived mycolic acids in *Mycobacterium* spp. cell walls [135]. Recently, a transcriptomic analysis of *Cryptosporidium parvum* has shown coregulation patterns between PKS/FAS genes with oocyst wall proteins, as well as trehalose and amylopectin metabolism, potentially supporting this hypothesis [136]. The metabolites from these complex PKS/FAS genes have remained difficult to identify, likely due to limitations in culturing and convolution with host background metabolites, however, with recent advances in genetics [137] and culturing improvements [136], the discovery of apicomplexan polyketide metabolites is a feasible task.

## 6. Outlook and Conclusions

Vast chemical space exists outside of prokaryotes, fungi, and plants. Elucidating chemical structures and their biosyntheses in less-studied natural product sources is a laborious task. The inherent difficulties in the collection and culturing of cells, lack of genetic and transcriptomic data, and insufficient genetic and biochemical tools all present obstacles to success in this area. Herein, we have illustrated pioneering work to connect eukaryotic metabolites with their cognate BGCs and summarize the creative techniques used to do so. Many examples have investigated BGCs of metabolites with known structures, some of which have been known for over half a century. These examples epitomize the importance of high-quality genomic and transcriptomic data and demonstrate the power of state-of-the-art sequencing technologies. With continued technological advances and genome sequencing efforts, like the Earth BioGenome Project that aims to sequence the genomes of all of Earth’s eukaryotic biodiversity over the next ten years [14] the future outlook for eukaryotic natural product discovery is exciting.

Accessing vast chemical space in bacteria and fungi has been greatly enhanced by genome mining efforts and heterologous expression technologies [11,138]. Perhaps heterologous expression platforms used for fungal eukaryotes [139] will be readily translatable in non-fungal eukaryotes, which is supported by the fact that multiple vertebrate PKS genes have been successfully expressed in fungal hosts [104,124]. These approaches can provide important structural information about molecules that complement metabolomics approaches to reveal the fully tailored secondary metabolites. Fortunately, heterologous expression platforms and genome editing tools are being continually improved, both of which will aid genetics-guided natural product discovery in phylogenetically diverse eukaryotes.

Goals of natural product research are to provide new chemical compounds with diverse bioactivities, advance the understanding of biology and chemistry, and improve the ability of researchers to access important molecules. As observed with platensimycin from bacteria or with kainic acid from seaweed, natural product biosynthesis can directly yield synthetically useful enzymes to facilitate assembly of complex molecules. As we continue to explore the bounds of nature’s biosynthetic repertoire, enzymes from phylogenetically diverse sources can aid in scientists’ ability to assemble important chemical structures.
